# I Hear You, but Do I Understand? The Relationship of a Shared Professional Language With Quality of Care and Job Satisfaction

**DOI:** 10.3389/fpsyg.2019.01310

**Published:** 2019-06-04

**Authors:** Manuel Stühlinger, Jan B. Schmutz, Gudela Grote

**Affiliations:** ^1^Department of Management, Technology and Economics, ETH Zurich, Zurich, Switzerland; ^2^Department of Communication Studies, Northwestern University, Evanston, IL, United States

**Keywords:** shared language, relational coordination, interprofessional collaboration, teamwork, psychological safety, quality of care, job satisfaction, patient safety

## Abstract

In various industries, individuals from different professions have to work together in a team to achieve their collective goal. Having gone through different educations, team members speak different professional languages, which poses a challenge to communication, and coordination in interprofessional teams. A shared language is believed to improve collaboration. In this study, we examine if a shared language in interprofessional healthcare teams is associated with better relational coordination and if both are connected to higher quality of care as well as job satisfaction of the staff. We shed light on possible mechanisms between shared language, and quality of care and job satisfaction, respectively, investigating relational coordination and psychological safety as mediators. We surveyed 197 healthcare workers (HCWs) from different professions in three rehabilitation centers in Switzerland. Multiple regression analyses showed that shared language was positively related to perceived quality of care and job satisfaction. Moreover, we found evidence for a serial mediation of these relationships by relational coordination and psychological safety. We discuss implications for healthcare and other types of interprofessional teams.

## Introduction

Every year, a large number of people die in hospitals because of preventable adverse events. Estimations for the United States vary from 44,000 to 98,000 deaths (Kohn et al., 2000) up to 440,000 deaths per year that preventable adverse events contribute to (James, 2013). In various other countries around the world, comparable incidences are reported (e.g., Vincent et al., 2001; Davis et al., 2002; Aranaz-Andres et al., 2008; Zegers et al., 2009). Given that communication errors are found to be a root cause of many adverse events (e.g., Morris et al., 2003; Leonard et al., 2004; Lingard et al., 2004) such as medication error or delayed treatment (Rabøl et al., 2011), we can assume that several thousand patients die in the United States alone every year because of inadequate communication in interprofessional healthcare teams.

Today, interprofessional teams are used in a variety of industries, because work gets more and more complex and therefore, requires a wide range of knowledge and skills that cannot be provided by one profession alone (Fay et al., 2006). This is especially true for the healthcare sector, where interprofessional collaboration between nurses, physicians, and other healthcare workers (HCWs) is required. Having run through different educational paths, the members of these teams often develop different values, beliefs, attitudes, and behaviors (Hall, 2005). This includes differences in understanding of patient conditions and treatments and in terminology. Put simply, each profession develops its own language (Frank, 1961; Hall, 2005). This is illustrated by the finding that rehabilitation staff, when presented with a description of a patient and her performance in cognitive tests, labeled her cognitive state anywhere from “normal for her age” to “severely impaired” (Wanlass et al., 1992). It is obvious that such differences in the use of basic terminology can lead to severe misunderstandings, which can slow down processes and potentially cause critical errors.

The importance of creating a shared language in interprofessional healthcare teams for avoiding communication-based errors has been recognized (e.g., Swayne, 1993; Pamplin et al., 2011). To promote the development of a shared language, interprofessional education is one option (Priddis and Wells, 2011), although interprofessional education itself needs a shared language at its base (e.g., Thistlethwaite et al., 2014; de Vries-Erich et al., 2017). Another option is to introduce classification systems which provide clear guidance on the use of medical terminology and therefore, help to establish a shared language among HCWs. One example is the International Classification of Functioning, Disability, and Health (ICF) by the World Health Organization [WHO] (2001). The ICF is a manual which provides HCWs with a framework to define and evaluate disabilities and functions of patients by using common classifications and codes (World Health Organization [WHO], 2001). For example, the ICF defines what a body function is and differentiates between different types of functions such as voice and speech functions. Speech functions are further divided into different aspects like fluency and speed of speech. With these classifications and definitions the ICF provides HCWs with a universal language to discuss patients’ insufficiencies and needs (Jette, 2006). The World Health Organization [WHO] (2013) states about the ICF that using a shared language makes collaboration between people from different professions more efficient.

Despite common belief that a shared language has positive effects on collaboration in interprofessional healthcare teams, quantitative research on the topic is scarce. Intervention studies have evaluated some of the instruments which are supposed to affect shared language (e.g., Cheung et al., 2012). However, to our knowledge, no study has quantitatively investigated whether a shared language actually has the anticipated positive consequences. Closest to this, one study has found a positive relationship between social capital (i.e., social resources available to a person, Coleman, 1988) and relational coordination, where shared language is a part of the cognitive dimension of social capital (Lee, 2013).

Our study tests the assumption that shared language is associated with better collaboration in interprofessional healthcare teams. We further examine if shared language is related to patient and staff outcomes (i.e., quality of care and job satisfaction) and we look at potential intermediate mechanisms, namely relational coordination and psychological safety.

With our study, we contribute to research and practice in two ways. First, our study empirically tests the relationship of a shared language in interprofessional healthcare teams with patient safety and staff outcomes. We aim to advance the conversation about whether striving for a shared language through the implementation of instruments like the ICF is worthwhile. Second, we help to improve the understanding of how shared language is related to patient and staff outcomes by suggesting possible mediating mechanisms. These insights could offer decision-makers potential starting points for interventions to improve team collaboration, overall quality of care, and job satisfaction. We hope our results prove instrumental for the prevention of communication errors which still cost lives every day.

## Theory and Hypotheses

### Effect of Shared Language and Mediation Through Relational Coordination

We are interested in understanding whether and how shared language is associated with quality of care and job satisfaction. We assume positive relationships between shared language and these outcomes. Furthermore, we hypothesize that these relationships operate through improved collaboration captured by the concept of relational coordination. Relational coordination comprises two basic components: communication quality and relationship quality among groups of people working together (Gittell, 2006). Communication quality includes frequent, timely, accurate, and problem-solving focused communication (Gittell et al., 2008a). Relationship quality includes shared goals, shared knowledge, and mutual respect (Gittell, 2006).

We suggest that a shared language (e.g., using the same medical expressions and definitions) improves both, communication and relationship quality, within the interprofessional healthcare team in several ways. For example, a shared language facilitates communication between HCWs by promoting a shared understanding of patient conditions and their demands. If HCWs can resort to the same terminology, this reduces the potential for misunderstanding and error, thereby increasing communication accuracy.

The relationship quality should also improve. For example, based on Nahapiet and Ghoshal’s (1998) take on social capital theory, shared language acts as a medium of social interaction through which members of an interprofessional team can exchange and combine knowledge. This leads to shared knowledge among the team members.

Previous studies have shown that relational coordination is related to increased quality of care (e.g., Gittell et al., 2000; Havens et al., 2010; Cramm and Nieboer, 2012) and higher job satisfaction of team members (Gittell et al., 2008a). High-quality communication allows team members to perform better. For example, frequent and timely communication gives team members more opportunities to update other team members regarding their actions, plans, and unexpected events, allowing them to adapt to the new situation. Also, fewer communication failures should occur, eventually translating into a better care quality (Williams et al., 2010). Furthermore, high-quality relationships, characterized by shared goals, shared knowledge and mutual respect, positively affect job satisfaction (Gittell et al., 2008a). Therefore, we hypothesize that shared language is positively related to quality of care and job satisfaction and that relational coordination in interprofessional healthcare teams acts as a mechanism for the two relationships.

Hypothesis 1: Shared language between HCWs is positively associated with (a) quality of care and (b) HCWs’ job satisfaction.
Hypothesis 2: Relational coordination between HCWs mediates the relationships of shared language between HCWs with (a) quality of care and (b) HCWs’ job satisfaction.

### Mediation Through Psychological Safety

We argue that psychological safety mediates the relationships of relational coordination with quality of care and job satisfaction. Psychological safety can be described as team members’ belief that their team is safe to take interpersonal risks (e.g., to speak up about issues), without fearing negative reactions or consequences by the other members (Edmondson, 1999).

Relational coordination incorporates high-quality relationships and communication, which should foster psychological safety in interprofessional teams. According to social exchange theory, reciprocal exchange evoke trust between the exchanging individuals (Blau, 1964). Relational coordination, including mutual respect, knowledge sharing, frequent, and timely communication, is characterized by positive, reciprocal social exchange between members from the interprofessional team and should therefore promote an open and trusting working climate (Carmeli and Gittell, 2009). Social exchange also reduces uncertainty regarding the behavior of other team members, thereby diminishing their fear of unforeseen negative reactions by other team members (Siemsen et al., 2009). This suggests that relational coordination is positively associated with psychological safety, where team members feel safe to challenge the status quo by speaking about errors that occurred or making suggestions for improvement. Supporting our assumption, psychological safety has been found to mediate the relationship between high-quality relationships (operationalized by relational coordination’s relationship qualities) and learning from failures (Carmeli and Gittell, 2009).

Psychological safety, in turn, has been found to be positively associated with a number of desirable outcomes such as speaking up (e.g., Detert and Burris, 2007; Walumbwa and Schaubroeck, 2009; Liang et al., 2012; Bienefeld and Grote, 2014), learning behaviors (e.g., Edmondson, 1999; Carmeli and Gittell, 2009; Hirak et al., 2012), and performance (e.g., Baer and Frese, 2003; Kessel et al., 2012). We expect that these positive outcomes translate into increased quality of care in the healthcare context. For example, speaking up is considered an important factor in preventing medical errors (Okuyama et al., 2014). Psychological safety should therefore, help to improve quality of care.

Psychological safety also enables an open team atmosphere (Edmondson, 1999). This climate is beneficial for the employees’ well-being (Kark and Carmeli, 2009) and is associated with the confidence that even when taking interpersonal risks, team members will not react negatively (Edmondson, 1999). Moreover, psychological safety is negatively related to conflict frequency in a team (Bunderson and Boumgarden, 2010). Therefore, we expect that in a team with high psychological safety, members feel more comfortable working together, which improves team members’ job satisfaction. These thoughts taken together, we hypothesize that relational coordination within interprofessional healthcare teams is positively related to quality of care and job satisfaction and that psychological safety acts as a mechanism for the two relationships.

Hypothesis 3: Psychological safety mediates the relationships of relational coordination between HCWs with (a) quality of care and (b) HCWs’ job satisfaction.

**FIGURE 1:**
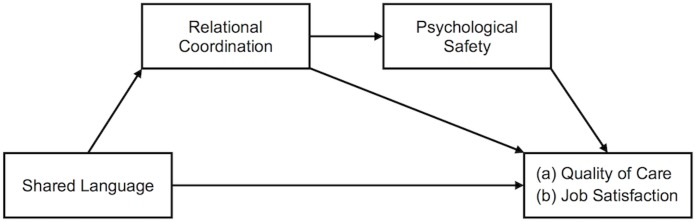
Serial mediation model showing the effect of shared language on (a) quality of care and (b) job satisfaction mediated by relational coordination and psychological safety.

Combining the above-stated hypotheses leads us to a serial mediation model, where shared language is positively related to relational coordination within interprofessional healthcare teams, which in turn is positively related to psychological safety, which in turn is positively related to quality of care and job satisfaction (see Figure 1).

Hypothesis 4: Relational coordination between HCWs and psychological safety serially mediate the relationships of shared language between HCWs with (a) quality of care and (b) HCWs’ job satisfaction.

## Materials and Methods

### Sample and Procedure

We gathered data from three Swiss rehabilitation centers. All three centers had, either recently or some time ago, implemented the ICF framework. The ICF was intended to facilitate the development of a shared language across professions. We expect that the development would only gradually be realized, therefore creating variance in the degree of shared language across professions. This created the opportunity to test the impact of shared language on the hypothesized outcomes, and the path through which shared language impacts those outcomes. We contacted currently employed HCWs from the three centers and encouraged them to complete our questionnaire either online or on paper.

In total, 237 employees started the questionnaire, of which we excluded 40 participants (16.9%) because their data was missing either for all (*N* = 29) or some (*N* = 11) of our model constructs completely, resulting in a final sample of 197 participants. Where we had item missing data, we used the remaining item data for scale means (Newman, 2014). From the final sample, 79 participants (40.1%) worked at rehabilitation center A, 59 participants (29.9%) worked at center B, and 59 participants (29.9%) worked at center C. We registered a response rate of 19.3% for center A and a response rate of 67.8% for center C.^1^

The final sample of 197 consisted of 150 women (76.1%) and 46 men (23.4%) with one participant not answering the question. The average age was 38.49 years (*SD* = 10.27), ranging from 22 to 66 years. Most participants were Swiss (71.1%) followed by German (20.3%), French (2.0%), Dutch (2.0%), Austrian (1.5%), and other nationalities (3.1%). Most participants were nurses and other care workers (55.8%) followed by occupational, physical and speech therapists (26.4%), social and psychological workers (7.6%), physicians (6.1%) and administrative staff (3%) with 2 participants not answering this question. Respondents from the nursing profession were slightly over- and the physicians under-represented. On average the participants had worked for 13.06 years (*SD* = 9.57) in their jobs and for 7.11 years (*SD* = 6.69) in their current institution. Table 1 provides a summary of sample characteristics segmented by study site.

**Table 1:** Sample characteristics segmented by rehabilitation centers.

Characteristic	Center A (*n* = 79)	Center B (*n* = 59)	Center C (*n* = 59)	Total (*N* = 197)
Age	37.08 (9.15)	38.44 (10.92)	40.47 (10.90)	38.49 (10.27)
Job tenure	11.83 (8.17)	12.86 (9.78)	14.93 (10.88)	13.06 (9.57)
Organizational tenure	6.32 (5.43)	6.30 (6.47)	8.99 (8.02)	7.11 (6.69)
Gender (% female)	78.5	66.1	83.1	76.5
Nationality (% non-Swiss)	30.4	45.8	10.2	28.9
Occupation				
Nursing staff	68.4	55.9	39.0	56.4
Therapists	13.9	20.3	49.2	26.7
Social care staff	6.3	10.2	6.8	7.7
Physicians	6.3	8.5	3.4	6.2
Administrative staff	2.5	5.1	1.7	3.1

*Means with standard deviations in parentheses are presented for variables age, job tenure, and organizational tenure; percentages are presented for all other variables.*

### Measures

#### Shared Language

Healthcare workers reported the degree to which a shared language is used with different professions. The measure was constructed to be similar to the measure of relational coordination (Gittell, 2002). Participants were asked “All in all, how uniformly do the members of the following professions speak a shared language?” and rated the degree of shared language with each of the professional groups that are involved in the rehabilitation process of the institution (e.g., physicians, nurses and other care staff, occupational therapists, and social workers) separately. The mean of all the ratings was used for data analysis. The response scale ranged from 1 = *not at all* to 5 = *completely.*

#### Relational Coordination

Relational coordination was measured using the items from Gittell (2002). They were slightly adapted to better suit our setting, e.g., by referring to “the patient” instead of “the status of joint replacement patients.” Five of the seven relational coordination qualities were included in our survey: mutual respect, shared knowledge, shared goals, communication frequency, and communication timeliness. The remaining two qualities, communication accuracy and problem-solving communication, were dropped due to constraints regarding the length of the questionnaire. Similarly to Gittell et al. (2008a), who also assessed only two communication qualities, we assume that the overall score still represents the relational coordination construct.

Similar to shared language, participants rated each relational coordination quality with regard to each of the professional groups involved in the rehabilitation process. Two sample items were: “All in all, how frequently do you communicate with the members of the following professions?” for communication frequency, and “All in all, how much do the members of the following professions respect you and the work you do with the patient?” for mutual respect. The two communication qualities included two ratings per profession, one referring to *ad hoc* communication, the other referring to communication using official communication platforms. Since we were interested in all communication channels, we averaged the two ratings. Answers were measured on a 5-point scale with anchors that matched the corresponding quality (α = 0.81).

To arrive at an overall relational coordination score, consistent with previous research (e.g., Gittell et al., 2008b; Havens et al., 2010), we first averaged the ratings for each quality, and then computed the mean of the five quality scores. Principal component analysis showed that the five quality scores yielded one factor with an eigenvalue of 2.86 and factor loadings for all dimensions ≥0.73.

#### Psychological Safety

We used four items by Edmondson (1999) to assess psychological safety. A sample item was: “All members of the team are able to bring up problems and tough issues.” The items were assessed with regard to the interprofessional team and the own profession. For our analysis, we used the mean of all eight ratings. The response scale ranged from 1 = *strongly disagree* to 5 = *strongly agree* (α = 0.73).

#### Job Satisfaction

To measure overall job satisfaction, we used one item similar to the one used by Scarpello and Campbell (1983). The item read: “All in all, I am very satisfied with my job.” Previous research showed that for global or overall job satisfaction, a single-item measure is appropriate, and has good psychometric properties (Scarpello and Campbell, 1983; Wanous et al., 1997; Wanous and Hudy, 2001). The response scale ranged from 1 = *strongly disagree* to 5 = *strongly agree.*

#### Quality of Care

We assessed quality of care with self-developed items based on the definition of quality care by Brook et al. (2000). As postulated by Brook et al. (2000), the two most important aspects of high quality of care are technical quality and respect toward patients. High technical quality means that treatments are performed in a professional way and based on the latest knowledge. Respectful treatment means to allow patients to participate in decisions regarding their therapy and, for example, incorporating their goals and obtaining their approval regarding the treatment. Quality of care was measured using 6 items: “The benefit of every treatment is evident”; “All treatments are performed in a professional way”; “All treatments are in accordance with the newest state of knowledge”; “The goals of the patient are considered when setting objectives”; “We take it very seriously that the patient agrees to the treatment objectives”; “In my opinion, the rehabilitation quality is very good here.” As with all the other measures, the items were rated by the HCWs and not the patients. The response scale ranged from 1 = *strongly disagree* to 5 = *strongly agree* (α = 0.85).

#### Control Variables

We included participants’ affiliation to rehabilitation centers as a control variable, because we expected differences between centers for all our model variables. To control for affiliation to rehabilitation center we used dummy coding with the center with the highest subsample, center A, as reference group.

### Confirmatory Factor Analyses

Since all model constructs were rated by HCWs, we conducted a confirmatory factor analysis (CFA) to test if the items used to measure the constructs load on corresponding latent factors. We conducted the CFA using R 3.5.0 (R Core Team, 2017) and the R package lavaan (Rosseel, 2012), and specified a five-factor model including all items used to measure shared language, relational coordination, psychological safety, quality of care, and job satisfaction. For psychological safety, we used four parcels, each incorporating the same item asked with regard to the two different reference groups (i.e., interprofessional team and own profession). Four cases (2.5%) of our total sample of 197 had missing on at least one of the items or parcels and were excluded for this analysis. Results showed that all items loaded significantly on the corresponding latent factors (*ps* < 0.001) and our measurement model had a reasonable model fit overall: χ^2^(111) = 227.48, *p* < 0.001; comparative fit index (CFI) = 0.90; root mean square error of approximation (RMSEA) = 0.07, 90% confidence interval (CI) [0.06, 0.09]; standardized root mean square residual (SRMR) = 0.05. This five-factor model had a significantly better fit than a one-factor model, where all items and parcels loaded onto one single latent factor: χ^2^(119) = 386.21, *p* < 0.001; CFI = 0.77; RMSEA = 0.11, 90% CI [0.10, 0.12]; SRMR = 0.08; Δχ^2^ = 158.72, *p* < 0.001.

## Results

### Data Analysis

We used SPSS 24 for data analysis. Intercorrelations, means, and standard deviations for key study variables are presented in Table 2. To test our hypotheses, we conducted multiple regression analyses with Hayes’ (2013) PROCESS macro for SPSS. Mediation was tested with PROCESS by computing an indirect effect and constructing a 95% bootstrap CI using 10,000 bootstrapped samples (Preacher and Hayes, 2008). We tested separate models for each of our hypotheses and our two outcome variables, quality of care, and job satisfaction. For missing data, we used pairwise deletion. All significance tests were two-tailed.

**Table 2:** Intercorrelations, means, and standard deviations for key study variables.

Measure	*M*	*SD*	*1*	*2*	*3*	*4*	*5*	*6*
1. Center B^a^	0.30		–					
2. Center C^a^	0.30		−0.43^∗∗∗^	–				
3. Shared language	3.79	0.64	0.16^∗^	0.23^∗∗^	–			
4. Relational coordination	3.67	0.41	0.20^∗∗^	0.16^∗^	0.59^∗∗∗^	–		
5. Psychological safety	4.03	0.60	0.03	0.23^∗∗∗^	0.37^∗∗∗^	0.42^∗∗∗^	–	
6. Quality of care	4.04	0.59	0.20^∗∗^	0.26^∗∗∗^	0.51^∗∗∗^	0.54^∗∗∗^	0.51^∗∗∗^	–
7. Job satisfaction	4.34	0.69	0.18^∗^	0.14^∗^	0.26^∗∗∗^	0.31^∗∗∗^	0.46^∗∗∗^	0.57^∗∗∗^

*Ns range from 194 to 197 due to missing data. ^a^Affiliation to rehabilitation center was dummy coded with center A as reference group, 1 indicates affiliation to that center and 0 indicates affiliation to one of the other two centers. ^∗^p < 0.05, ^∗∗^p < 0.01, and ^∗∗∗^p < 0.001.*

### Test of Hypotheses

Hypothesis 1 proposed that a shared language between HCWs is positively associated with (a) quality of care and (b) HCWs’ job satisfaction. We found significant total effects of shared language on quality of care (β = 0.41, *p* < 0.001) and on job satisfaction (β = 0.17, *p* = 0.018). Therefore, Hypotheses 1a and 1b were supported.

Hypothesis 2 predicted that the relationships of shared language among HCWs with (a) quality of care and (b) HCWs’ job satisfaction will be mediated by relational coordination among HCWs. We found a significant indirect effect of 0.17, 95% CI [0.09, 0.26], from shared language through relational coordination (β = 0.54, *p* < 0.001) to quality of care (β = 0.33, *p* < 0.001). Similarly, we found a significant indirect effect of 0.12, 95% CI [0.01, 0.25], from shared language through relational coordination (β = 0.54, *p* < 0.001) to job satisfaction (β = 0.20, *p* = 0.020). Thus, Hypotheses 2a and 2b were supported.

Hypothesis 3 proposed that psychological safety mediates the relationships of relational coordination between HCWs with (a) quality of care and (b) HCWs’ job satisfaction. We found a significant indirect effect of 0.17, 95% CI [0.09, 0.28], from relational coordination through psychological safety (β = 0.39, *p* < 0.001) to quality of care (β = 0.30, *p* < 0.001). Similarly, we found a significant indirect effect of 0.25, 95% CI [0.14, 0.41], from relational coordination through psychological safety (β = 0.39, *p* < 0.001) to job satisfaction (β = 0.39, *p* < 0.001). Therefore, Hypotheses 3a and 3b were supported.

**FIGURE 2:**
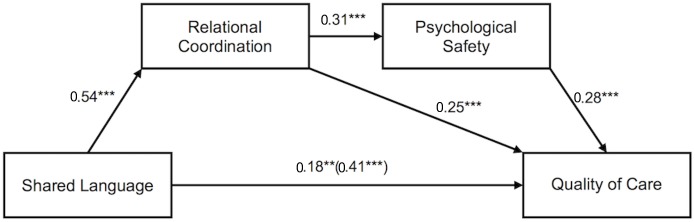
Serial mediation model showing the effect of shared language on quality of care mediated by relational coordination and psychological safety. Values depicted are standardized regression coefficients. The total effect of shared language on quality of care is written in parentheses. ^∗∗^*p* < 0.01. ^∗∗∗^*p* < 0.001.

Hypothesis 4 predicted that relational coordination between HCWs and psychological safety serially mediate the relationships of shared language between HCWs with (a) quality of care and (b) HCWs’ job satisfaction. As depicted in Table 3 and Figure 2, We found a significant indirect effect of 0.04, 95% CI [0.02, 0.09], from shared language through relational coordination (β = 0.54, *p* < 0.001), next through psychological safety (β = 0.31, *p* < 0.001) to quality of care (β = 0.28, *p* < 0.001). Controlling for the two mediators reduced the total effect of shared language on quality of care (β = 0.41, *p* < 0.001) to a still significant direct effect (β = 0.18, *p* = 0.008). Thus, Hypothesis 4a was supported.

**Table 3:** Multiple linear regression analyses for the serial mediation models predicting quality of care and job satisfaction.

					Quality of care				Job satisfaction			
	Relational coordination		Psychological safety		Model 1		Model 2		Model 1		Model 2	
Predictor	β	*p*	β	*p*	β	*p*	β	*p*	β	*p*	β	*p*
Center B (vs. A)	0.16	0.018	0.01	0.856	0.25	0.000	0.19	0.002	0.24	0.003	0.20	0.007
Center C (vs. A)	0.10	0.122	0.15	0.040	0.27	0.000	0.19	0.003	0.20	0.011	0.12	0.100
Shared language	0.54	0.000	0.15	0.058	0.41	0.000	0.18	0.008	0.17	0.018	0.01	0.929
Relational coordination			0.31	0.000			0.25	0.000			0.08	0.329
Psychological safety							0.28	0.000			0.39	0.000
R^2^	0.37		0.22		0.33		0.46		0.12		0.26	
F	37.36	0.000	13.88	0.000	31.23	0.000	32.4	0.000	8.37	0.000	13.12	0.000

*N = 197. β values are standardized regression coefficients.*

**FIGURE 3:**
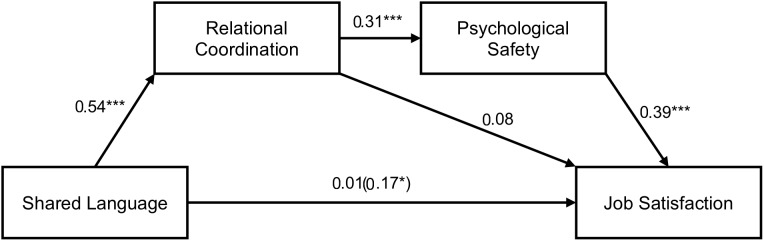
Serial mediation model showing the effect of shared language on job satisfaction mediated by relational coordination and psychological safety. Values depicted are standardized regression coefficients. The total effect of shared language on job satisfaction is written in parentheses. ^∗^*p* < 0.05. ^∗∗∗^*p* < 0.001.

Similarly, as depicted in Table 3 and Figure 3, we found a significant indirect effect of 0.07, 95% CI [0.03, 0.13] from shared language through relational coordination (β = 0.54, *p* < 0.001), next through psychological safety (β = 0.31, *p* < 0.001) to job satisfaction (β = 0.39, *p* < 0.001). Controlling for the two mediators reduced the total effect of shared language on job satisfaction (β = 0.17, *p* = 0.018) to a non-significant direct effect (β = 0.01, *p* = 0.929). Therefore, Hypothesis 4b was supported.

## Discussion

The main goal of our study was to examine the role of shared language in the context of interprofessional collaboration in healthcare. To test the assumption of the ICF and other classification systems that a shared language among HCWs is vital, we investigated the relationship of shared language with important outcomes and underlying mechanisms. The results support our proposed models. We found a positive relationship of shared language with quality of care and job satisfaction. Both of these relationships were serially mediated by relational coordination and psychological safety.

The results indicate that the relationship with quality of care as the outcome was partially mediated whereas the relationship with job satisfaction was fully mediated. This suggests that in the case of quality of care, shared language explains variance which is not captured by relational coordination or psychological safety. One possibility is that shared language directly leads to fewer communication errors. For example, using the same terminology should eliminate certain types of communication problems, which might not be captured by relational coordination’s communication qualities, therefore exerting a direct effect on quality of care. In contrast, our two tested mediators explain most of the shared variance between shared language and job satisfaction, rendering the direct effect non-significant. Therefore, we may have caught the most important mediators with relational coordination and psychological safety.

Overall, our findings indicate that a shared language between HCWs is associated with better interprofessional collaboration in healthcare teams and with higher quality of care as well as higher job satisfaction of HCWs. Psychological safety in those interprofessional teams seems to play an important role in mediating these effects.

### Theoretical Implications

Our study contributes to the existing literature in three ways. First, it provides quantitative evidence for a positive relationship between shared language, relational coordination, and beneficial outcomes, namely quality of care and job satisfaction. Previous literature on the potential positive effects of shared language in the healthcare sector has mainly used qualitative methods (e.g., Cedraschi et al., 1998; Cheung et al., 2012) without directly addressing the relationship between shared language and quality of care or job satisfaction. Our study therefore extends previous literature with quantitative and more specific support for the assumed beneficial effects of shared language.

Second, with shared language we have identified a valuable antecedent of relational coordination, extending the existing theory. Previous studies have explored a number of antecedents, for example, supervisory span (Gittell, 2001), high performance work practices (Gittell et al., 2010), boundary spanners, and team meetings (Gittell, 2002). However, this study is the first to explore the impact of shared language on relational coordination. Given the strong relationship between them, we think shared language is an important antecedent and might even be a prerequisite for developing relational coordination.

Third, we shed light on the mechanisms that act between shared language and outcomes. Besides relational coordination we found that psychological safety mediates said relationships. Based in part on previous findings (Carmeli and Gittell, 2009), we proposed psychological safety to be a mechanism between relational coordination, and our outcome variables. Whereas Carmeli and Gittell (2009) solely looked at the relationship quality of relational coordination, we considered the whole relational coordination construct, including communication quality. Communication quality, which includes frequent, timely, accurate, and problem-solving focused communication, is critical for a successful coordination in care. This is illustrated by the finding that communication failures (e.g., due to poor timing or inaccurate information) can lead to inefficiency, delay, and even errors (Lingard et al., 2004). Therefore, our study advances the work of Carmeli and Gittell (2009) by including care-critical communication quality. Our results are consistent with the notion that relational coordination could help to foster psychological safety in teams, enriching our understanding of how psychological safety is created in teams and organizations.

### Limitations

A limitation of our study is that we cannot rule out that the chain of causality between shared language, relational coordination, psychological safety, and quality of care and job satisfaction is different. For example, it is possible that a psychologically safe climate in an interprofessional team promotes the development of relational coordination, because in psychologically safe teams, individuals feel safer to share information, and knowledge (Kessel et al., 2012). Theoretically, one could also argue for a mutually enforcing relationship between relational coordination and psychological safety. To gain certainty over the causal processes, longitudinal data is necessary, with which developmental aspects can be captured.

Gathering all data from one source (i.e., the HCW) can lead to common method bias, resulting in statistically inflated or deflated observed relationships (Podsakoff et al., 2003). Yet, we sought to reduce the common method bias through psychological separation (Podsakoff et al., 2003) by using different response formats for shared language and relational coordination (items referring to each professions) on the one hand, and psychological safety, quality of care, and job satisfaction (items referring to team or general situation) on the other.

Finally, we used a new measure for quality of care. Our reliability test and CFA looked promising. However, to gain further confidence in the validity of this measure it should be tested in other samples and future studies should aim to include objective quality indicators (e.g., objective patient outcomes).

### Future Research on Shared Language

Future research could take a closer look at the construct of shared language and try to further dissect it. In our study, we provided positive results by measuring shared language in a general way. It would be interesting to see what is needed to create shared language and which specific elements constitute a successful shared language. A more detailed look at the term “shared language” and its elements could help to gain further insights into what exactly is necessary to improve interprofessional collaboration.

Moreover, the implementation of interprofessional education or of frameworks such as the ICF, which are aimed at creating a shared language, should generally be accompanied by quantitative research to test its success more objectively (e.g., Cheung et al., 2012). Such studies would allow to gain further insights into the efficacy and practicability of interventions targeted at shared language.

Our study provides first evidence that a shared language in interprofessional healthcare teams is associated with better performance in the form of quality of care. Future studies could try to replicate and further develop our model in other contexts. There is an increasing number of organizational contexts today, where employees with different professions or different educational backgrounds have to work together. We expect that shared language in interprofessional teams from other industries such as research or product development works in similar psychological mechanisms and therefore also leads to higher quality work and higher employee job satisfaction via increased relational coordination and psychological safety. We hope that our research can be the starting point of further studies investigating shared language in interprofessional teams from a variety of industries.

### Practical Implications and Conclusion

Our findings reinforce the calls for a shared language in the interprofessional healthcare sector (e.g., Swayne, 1993; Pamplin et al., 2011). The results provide quantitative support for the importance of shared language for interprofessional collaboration. Therefore, it is important to promote efforts to enhance shared language in interprofessional teams, for example by means of interprofessional education or the use of classification systems like the ICF.

Our findings also provide insights into possible mechanisms, through which a shared language may influence outcomes. For practitioners, these represent potential starting points for alternative interventions in areas, where a shared language might be very difficult to achieve. Supporting leaders in establishing relational coordination and psychological safety could be a possible course of action. For example, supportive (Edmondson, 1999) and inclusive (Nembhard and Edmondson, 2006) leaders who are open to and invite speaking up behavior have been shown to be associated with a psychologically safer climate.

In conclusion, we hope that our findings will engender a new research stream on shared language. Learning more about how shared language develops and what specific characteristics of shared language improves collaboration as well as psychological safety in interprofessional teams will contribute to enhance team performance and deliver better care. In healthcare, we believe that a new perspective and focus on shared language can help prevent at least some of the futile deaths caused by communication errors.

## Ethics Statement

For this study, we gathered survey data. Participants received written information about the study prior to answering the questions and had the opportunity to decline to participate in the study without any negative consequences. Participants gave their consent by continuing with the study either by clicking on a button (online version) or by filling in the questionnaire (paper version). No personal health-related or patient-related data were assessed. Furthermore, the survey was completely confidential and the data anonymized after completion of the study. Therefore, in compliance with Swiss national law, no ethics approval was necessary.

## Author Contributions

MS was responsible for developing the theoretical model, analyzing the data, and drafting and revising the manuscript. GG contributed to project planning. GG and JS contributed to drafting and revising the manuscript and approved the submitted version.

## Conflict of Interest Statement

The authors declare that the research was conducted in the absence of any commercial or financial relationships that could be construed as a potential conflict of interest.

## Footnotes

1 Since the HCWs were contacted via a contact person from within the center, we do not have the exact number of HCW who have been contacted for the study for center B. Therefore, we cannot provide a response rate for center B.
